# Notes on Veterinary Medicine in Great Britain

**Published:** 1885-01

**Authors:** N. S. Townsend

**Affiliations:** Professor of Agriculture, State University, Columbus, Ohio


### Art. II.—NOTES ON VETERINARY MEDICINE IN GREAT BRITAIN.

BY N. S. TOWNSEND, M.D.,

*Professor of Agriculture, State University, Columbus, Ohio.*

In a recent visit to the British Isles, the principal object of which was to ascertain the condition there of agricultural education, the writer had some opportunity to become acquainted with the state of veterinary science and practice, and to judge of the estimation in which the veterinary profession is held by stock owners and the public. Possibly a portion of your readers may be interested in the conclusions reached upon veterinary matters.

In nearly all parts of Great Britain there appears to be a sufficient number of able veterinary practitioners. In the main they are men of good education and of general scientific as well as professional training, consequently they appear to have good social standing, and to enjoy the confidence of the public. In many cases it was pleasing to see evidences of comity and mutual respect between physicians and veterinarians, and of a desire to share in efforts for the better sanitary condition of the people. The number and standing of veterinarians in Great Britain is doubtless to be explained in part by the high character of their veterinary schools. The veterinary college at Camden Town, near London, and the two colleges in the city of Edinburgh, all of which were visited, have complete equipments and very able teachers. Several opportunities of attending the examinations of veterinary students showed on their part praiseworthy determination to secure the same broad and scientific training that is understood to be necessary for the young physician. The number of associations for mutual improvement among veterinarians and the profitable character of their meetings, some of which the writer was permitted to attend, may also help to account for the good standing of the veterinary profession. Observations and investigations made by individuals, when presented and discussed at the meetings of an association and reported in veterinary periodicals, which all take, are then the common property of the profession and a benefit to all.

The owners of stock who were met with at the Royal Agricultural Society's Exhibition at Shrewsbury, at the Highland and Agricultural Society's Exhibition at Edinburgh, and at two exhibitions in Dublin, evidently appreciated the value of the assistance they so often require from veterinarians. This appreciation appeared to be due not alone to the amount of benefits received, but also to the general knowledge which stock-owners there possess of the nature, symptoms and effects of disease. In the houses of farmers it was not uncommon to meet with valuable veterinary books; some of these were old, such as Clater's, Blaine's, Youatt's, or Percivall's; but still oftener some of the later works were seen, such as those of

Fleming, Williams, Gamgee, Armitage, or Steele. The great advantage which farmers derive from a knowledge of animal diseases was seen in the promptness with which serious disease was recognized and professional help obtained, and in the less frequent resort to injurious quack nostrums.

Perhaps a word or two by way of application may be tolerated. In many parts of the United States outside of the large cities, educated veterinarians are not to be found, and stock-raisers living in the country often suffer loss in consequence. Does not this promise a profitable occupation for young men who are willing to make thorough preparation for veterinary practice? In many of the older States veterinarians are so few and so far between that they can derive but little stimulus to study or research. Is it not probable that a well-educated veterinarian would often be welcomed to membership in ordinary medical societies, and with mutual benefit, inasmuch as the medical and veterinary professions have so much in common? Perhaps it would pay some of our young country doctors, who are impatiently waiting for practice to take a winter's course in a veterinary college. They might afterwards make themselves more serviceable to their patrons, and it is not probable they would suffer in the estimation of sensible people if found always ready to relieve suffering, whoever or whatever the subject. Finally, will not a more thorough study of domestic animals, both in health and in disease—such as appears to have been made abroad—be good for the animals, profitable to their owners, and lead to a better appreciation of the veterinary practitioner?

